# Whole-exome sequencing revealed a likely pathogenic variant in *NF1* causing neurofibromatosis type I and Arrhythmogenic Cardiomyopathy

**DOI:** 10.1186/s12872-024-03878-z

**Published:** 2024-04-23

**Authors:** Maryam Pourirahim, Golnaz Houshmand, Leyla Abdolkarimi, Majid Maleki, Samira Kalayinia

**Affiliations:** 1 Cardiogenetic Research Center, Rajaie Cardiovascular Medical and Research Center, Iran University of Medical Sciences, Tehran, Iran; 2 Rajaie Cardiovascular Medical and Research Center, Iran University of Medical Sciences, Tehran, Iran

**Keywords:** Neurofibromatosis, Genetic, Variant, Cardiomyopathy, Arrhythmogenic cardiomyopathy

## Abstract

**Background:**

Neurofibromatosis type I (NF1) is a genetic disorder characterized by the tumor’s development in nerve tissue. Complications of NF1 can include pigmented lesions, skin neurofibromas, and heart problems such as cardiomyopathy. In this study, we performed whole-exome sequencing (WES) on an Iranian patient with NF1 to identify the genetic cause of the disease.

**Methods:**

Following clinical assessment, WES was used to identify genetic variants in a family with a son suffering from NF1. No symptomatic manifestations were observed in other family members. In the studied family, *in silico* and segregation analysis were applied to survey candidate variants.

**Results:**

Clinical manifestations were consistent with arrhythmogenic cardiomyopathy (ACM). WES detected a likely pathogenic heterozygous missense variant, c.3277G > A:p.Val1093Met, in the *NF1* gene, confirmed by PCR and Sanger sequencing. The patient’s parents and brother had a normal sequence at this locus.

**Conclusions:**

Although there is no cure for NF1, genetic tests, such as WES, can detect at-risk asymptomatic family members. Furthermore, cardiac evaluation could also help these patients before heart disease development.

**Supplementary Information:**

The online version contains supplementary material available at 10.1186/s12872-024-03878-z.

## Introduction

Neurofibromatosis (NF), with an incidence of 1 to 5 per 10 000 live births worldwide, is a genetic disorder that causes tumors in nerve tissue [1, 2]. The inheritance pattern of NF is autosomal dominant [2] and is divided into 3 categories: NF1, NF2, and schwannomatosis [3]. NF1, also called “von Recklinghausen’s disease”, is caused by germ-line mutations in tumor suppressor genes [4], with symptoms including pigmentary lesions (brown macules, freckles, skin folds, and Lisch nodules), skin neurofibromas, skeletal abnormalities (scoliosis, tibia pseudarthrosis, and orbital dysplasia), brain tumors (gliomas of the visual pathway and glioblastomas), and peripheral nerve tumors (spinal neurofibromas, plexiform neurofibromas, and malignant peripheral nerve sheath tumors) [4]. It typically manifests during childhood, although it can appear in adulthood as well. NF1 sometimes causes cardiovascular diseases [5], such as congenital heart diseases [6] and cardiomyopathies [7]. In a study by Ibarrola et al., left ventricular non-compaction and orthodromic atrioventricular tachycardia were observed in a patient with NF1 [8]. Moreover, a patient with NF1 and hypertrophic cardiomyopathy (HCM) was described by Jurko et al. [9]. Nguyen et al. [10] concluded that in patients with sizeable deletions of the *NF1* gene, HCM was more common. *NF1* gene, located on chromosome 17q11.2 and encodes a large cytoplasmic protein known as neurofibromin. Neurofibromin serves as a tumor suppressor and plays a critical role in the regulation of cellular signaling pathways, particularly the Ras/mitogen-activated protein kinase (MAPK) pathway. Treatment for NF1 focuses on managing symptoms and complications rather than curing the condition. In the present study, we performed whole-exome sequencing (WES) on an Iranian family with a son suffering from NF1 and arrhythmogenic cardiomyopathy (ACM) to identify the genetic cause(s). ACM is a genetic disorder characterized by the replacement of heart muscle tissue with fibrous or fatty tissue. It typically manifests in adolescence or young adulthood, although it can present at any age. Arrhythmias and chest pain are the common features of ACM. Genetic counseling and testing for affected NF1 and/or ACM individuals and their families to identify the mutation and assess the risk of passing it on to future generations. To our knowledge, this is the first report of ACM manifestation in a patient with NF1.

## Methods

### Study subject, clinical evaluation, and ethics statement

**Fig. 1:**
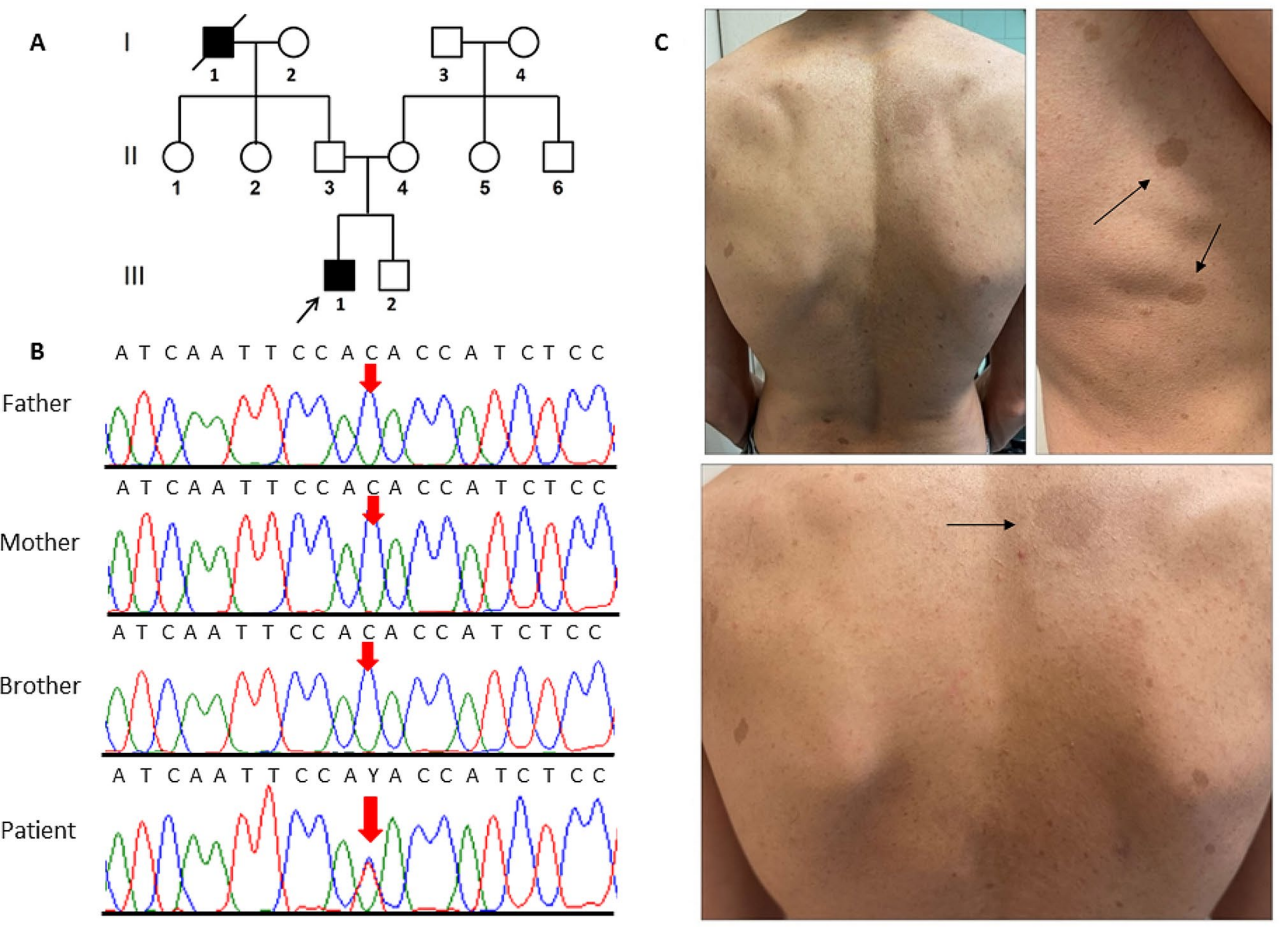
The image presents pedigree, sequencing chromatograms, and neurofibromatosis type 1 clinical manifestations in a family affected by *NF1* mutation. **(A)** The pedigree of an Iranian family with NF1. Only the proband (III-1) is affected (pointed with an arrow). **(B)** The snapshot of the sequencing reads: The proband (III-I) carries the c.3277G > A variant in a heterozygous status. The black arrow shows the location of the mutated nucleotide. **(C)** Clinical manifestations of the proband (III-1) with NF1 which includes multiple brown spots in different parts of the patient’s body

A 20-year-old male patient was referred to the Cardiogenetic Research Center, Rajaie Cardiovascular Medical and Research Center, Iran University of Medical Sciences, Tehran, Iran with a complaint of chest pain and arrhythmias. The patient’s cardiac symptoms started at 16 years old, and dark spots were observed on his body at 2 years old. He was the second child of non-consanguineous healthy parents (Fig. 1A). A comprehensive clinical workup, including echocardiography, magnetic resonance imaging, and physical examination, was performed. The results of the clinical investigations of the other family members were normal. For precise diagnosis, WES was done on the proband (Fig. 1A: III-2). The study was performed in accordance with the Helsinki Declaration and was approved by the Ethics Committee of Rajaie Cardiovascular Medical and Research Center, Iran University of Medical Sciences, Tehran, Iran (IR.RHC.REC.1402.017). Written informed consent was obtained from the family.

### WES and segregation analysis

Genomic DNA was extracted from the peripheral blood samples of the proband and his family members using the Roche kit (DNsol Mini kit of Roche: lot No. 500,021,091,210,050). WES was performed at Macrogen (Amsterdam, Netherlands) on the Illumina HiSeq 6000. Raw data were analyzed by Rajaie Cardiovascular Medical and Research Center, Tehran, Iran. BWA, v.07.17, was used to align the reads to the reference genome (NCBI, hg19) [11]. Single-nucleotide polymorphisms and insertions and/or deletions were called using GATK, v.4.1.4.1 [12]. All the determined variants were annotated by ANNOVAR [13]. Frequency databases, consisting of Iranome (http://www.iranome.ir), Greater Middle East (http://igm.ucsd.edu/gme/), 1000 Genomes Project (http://www.1000genomes.org), and Genome Aggregation Database (https://gnomad.broadinstitute.org), were drawn upon to filter the variants. The variants with a frequency of more than 1% in these databases were excluded. In the next step, synonymous, intronic, and intergenic variants were removed. Then, variants related to genes associated with the patient’s phenotype were retained. The pathogenicity of the variants was evaluated using prediction tools, composed of CADD (https://cadd.gs.washington.edu/home), SIFT (https://sift.bii.a-star.edu.sg/), PolyPhen-2 (http://genetics.bwh.harvard.edu/pph2/), PROVEAN (http://provean.jcvi.org/index.php), and MutationTaster (http://www.mutationtaster.org/). The results were interpreted based on the 2015 guidelines of the American College of Medical Genetics and Genomics (ACMG) [14]. The conservation analysis of the protein change position was performed by comparing the amino acid sequences of different species on PolyPhen-2.

Segregation analysis was carried out by polymerase chain reaction (PCR)-direct Sanger sequencing to evaluate the family members. For variant amplification, Primer F (5′-AGGTTTATTTGAGGGGAAGTG-3′) and Primer R (5′-ACTCAATGCCAACGTAGACAG-3′) were designed using Gene Runner, v. 6.5.52. PCR was performed (the denaturing stage: 30 s at 95 °C, the annealing stage: 30 s at 60 °C, and the extending stage: 30 s at 72 °C [35 cycles]) on a SimpliAmp (Thermo Fisher Scientific) with 100 ng DNA, 1.5 mmol/L of MgCl2, 200 mmol/L of dNTP, 10 pmol/L of the primers, and 1 U of Taq DNA polymerase (Amplicon, UK). The PCR products were sequenced with the ABI Sequencer 3500XL PE machine (Applied Biosystems) and analyzed using BioEdit, v.7.0.5.3.

## Results

### Clinical evaluation

**Fig. 2:**
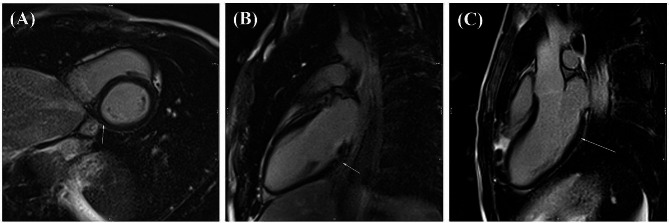
The late enhancement images at the basal level short-axis view and two-and three-chamber views, respectively, show subepicardial enhancement in the basal inferior, inferolateral walls of the left ventricle

**Fig. 3:**
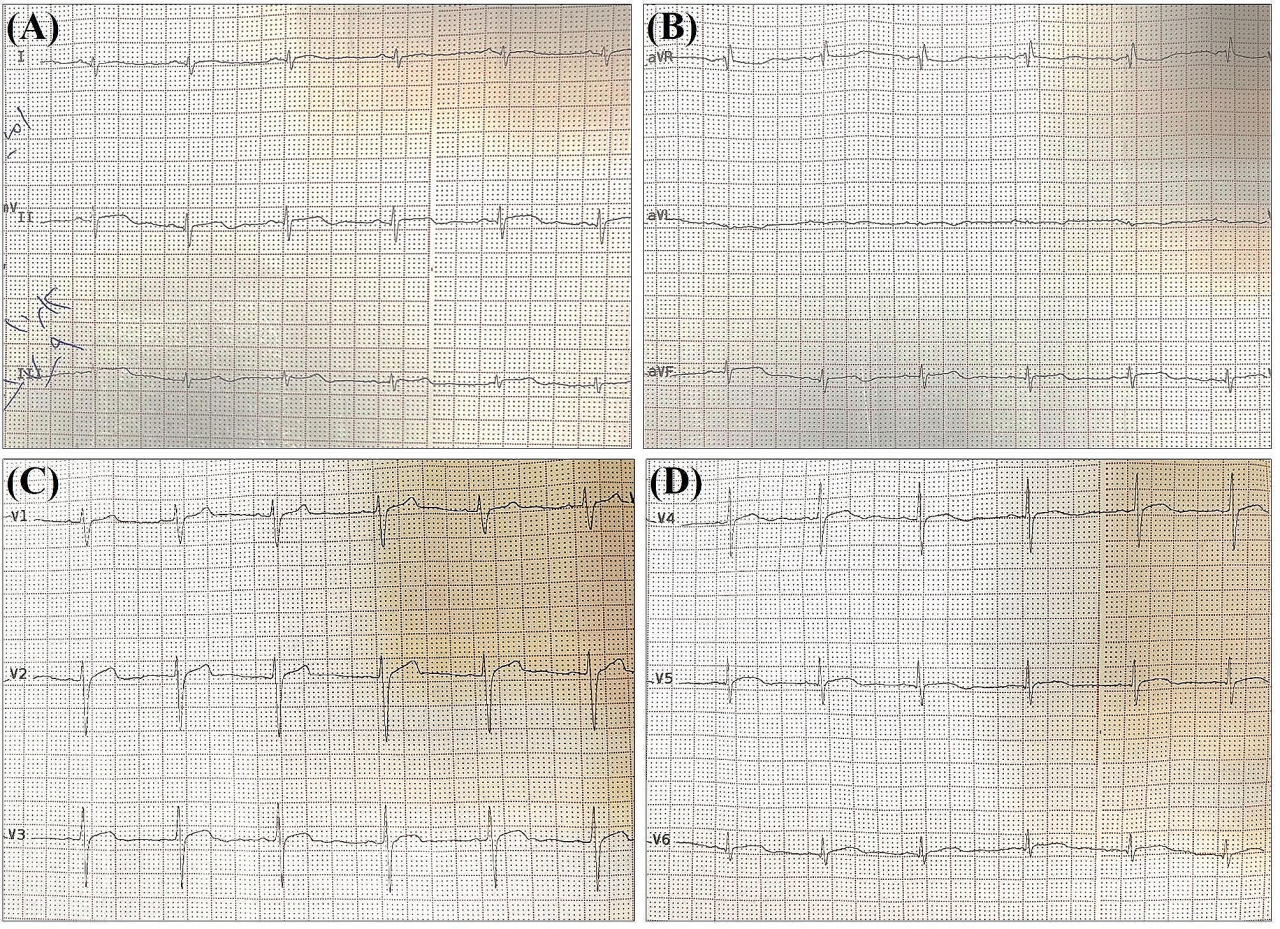
The 12-lead ECG showing rS in the inferior leads and persistent, prolonged S and poor R progression in the precordial leads. Right ventricular hypertrophy (RVH) was seen in the ECG

The physical examination of the proband (Fig. 1A: III-1) indicated poor eyesight and more than 6 flat and light brown spots (café-au-lait spots) on the skin of his legs, hands, back, chest, sides, armpits, neck, and abdomen (Fig. 1C). The patient had learning disability, especially with mathematics and reading. According to the description given by the family, hyperactivity disorder and speech delays were also observed in the patient’s childhood. Magnetic resonance imaging (Fig. 2A-C) and echocardiography (Fig. 3A-D) revealed right ventricular ACM. Cardiac magnetic resonance imaging showed a normal left ventricular size, a left ventricular end-diastolic volume indexed to the body surface area of 74 mL/m^2^, a moderately reduced left ventricular ejection fraction of 43%, a normal right ventricular size, an end-diastolic volume indexed to the body surface area of 65 mL/m^2^, and a mildly reduced right ventricular ejection fraction of 42%. Segmental dyskinesia was observed in the sub-tricuspid, mid-right ventricular free wall, and right ventricular outflow regions (Supplementary Videos 1–3). The short tau inversion recovery (STIR) sequences indicated no inflammation or edema. Late gadolinium enhancement sequences showed subepicardial fibrosis in the basal inferior and inferolateral walls of the left ventricle (Fig. 2). According to the cardiac magnetic resonance imaging features, consisting of biventricular systolic dysfunction, right ventricular segmental dyskinesia, and fibrotic patterns in the left ventricular myocardium, the phenotype was considered compatible with ACM with biventricular involvement.

### Molecular evaluation

WES detected a heterozygous variant in exon 25 of the *NF1* gene (NM_001042492.3: c.3277G > A:p.Val1093Met) in the proband (Fig. 1A: III-1), probably responsible for neurofibromatosis type I (NF1). Based on the ACMG classification (PM1, PM2, PM5, and PP5), c.3277G > A is a likely pathogenic variant. PCR and Sanger sequencing confirmed the c.3277G > A variant in the affected proband. The patient’s unaffected parents (Fig. 1A: II-3 and II-4) and brother (Fig. 1A: III-2) were normal for this locus (Fig. 1B). The missense variant was supported as the cause of the disease by CADD, SIFT, PolyPhen-2, PROVEAN, FATHMM, and GERP^++^ (Table 1).

**Table 1:** Identified variant in this study

Gene/transcript	Variant	^1^CADD	^2^SIFT	^3^PolyPhen-2	^4^PROVEAN	^5^FATHMM	^6^GERP++
*NF1* NM_001042492.3	c.3277G > A p.Val1093Met	28.7	0.04	0.9	-2.8	0.9	5.3

^1^ CADD, Phred ≤ 20: Natural; Phred > 20: Damaging

^2^ SIFT, score ≤ 0.05: Deleterious; score > 0.05: Tolerable

^3^ PolyPhen-2, score ≤ 0.15: Benign; score > 0.85: Damaging

^4^ PROVEAN, score ≤ -2.5: Deleterious; score > -2.5: Tolerable

^5^ FATHMM, score ≤ 0.5: Neutral; score > 0.5: Deleterious

^6^ GERP + + scores range from − 12.3 to 6.17, higher scores indicating conserve locus.

## Discussion

In our present study, we found a missense variant, c.3277G > A, in exon 25 of the *NF1* gene in the proband, probably responsible for his NF1 condition0. To our knowledge, our investigation is the first to report the association between the *NF1* variant and ACM. Previous studies have reported other cardiovascular manifestations secondary to *NF1* mutations, including congenital heart diseases, vasculopathy, and hypertension [15]. Dunning-Davies et al. [16] concluded that congenital heart diseases, especially pulmonary stenosis, were associated with, with frequencies ranging from 0.4 to 6.4%. NF1-associated vasculopathy, comprises stenosis, aneurysms, and arteriovenous malformations, the second cause of death worldwide, renal artery stenosis is the most common appearance, occurring in at least 1% of patients with NF1. Nonetheless, the clinical investigation of the proband did not detect vasculopathy. Hypertension is strongly associated with increased mortality in NF1 patients and should be screened annually [15]. Prior to our current study, hypertension was not observed in similar investigations, although this complication may occur in older age. In 2019, Ritter et al. [17] described a patient with isolated fetal HCM as the presenting feature of NF1. Left ventricular hypertrophy, a rare finding in NF1, is primarily reported in patients with 1.4 Mb *NF1* deletion. The loss of neurofibromin in cardiomyocytes causes cardiac hypertrophy and progressive HCM in *NF1* homozygous null mouse models, suggesting that adults with NF1 who have a normal heart structure in childhood might be at risk of cardiomyopathy later in life. Most likely, fetal cardiomyopathy, asymmetric septal hypertrophy, and subsequent diagnosis of NF1 are not coincidental. Nevertheless, given that the patient’s cardiac dysfunction in the study by Ritter et al. had no other causes, the most likely explanation is that cardiomyopathy was an early manifestation of left ventricular hypertrophy associated with NF1. The authors posited that adding *NF1* to diagnostic panels could allow early diagnosis of patients with NF1. In 2012, Alkindy et al. [18] assessed a 71-year-old father and his 29-year-old son who had symptoms of echocardiographically diagnosed HCM manifesting as cardiovascular-related complications of NF1. Echocardiographic findings of a ratio of the free wall of the septum to the left ventricular posterior wall of greater than 1.5 (indicative of HCM) are seen in at least 4% of patients with NF1. Cardiac hypertrophy is the most common risk factor in mortality and cardiovascular complications in these patients. Congenital heart diseases and cardiomyopathy are also common concomitants of several growth and development syndromes that affect RASopathies. In these syndromes, increased signaling through Erk1/2 and mTOR complex 1 causes cardiomyopathy. Neurofibromin regulates Ras signaling, and Ras activation causes progressive cardiac hypertrophy in adult mice. Our study patient was also suffering from cardiomyopathy, ACM. All such patients show NF1 pigmentary changes with very few NF1-related complications milder in terms of clinical phenotypes. Our patient had more than 6 light brown spots on the skin of his legs and upper body. In 2016, Jurko et al. [9] described an 18-year-old boy suffering from NF1 and HCM with forward systolic movements of the anterior leaflet of the mitral valve. The authors concluded that one of the common causes of severe arrhythmias and sudden deaths was HCM and that cardiac involvement caused significant problems in patients with NF, although relevant information was scarce. Patients with NF can develop progressive cardiomyopathy, hence the need for follow-ups in cardiology clinics to swiftly identify any change in the electrocardiogram or the chest X-ray. Additionally, the follow-up of HCM is necessary as it may require surgery [9]. This study holds significance in several aspects, clinical Correlation: The study found that the patient exhibited signs consistent with ACM, which is a significant complication associated with NF1. This correlation between the genetic variant and clinical presentation highlights the importance of genetic testing in diagnosing and managing NF1 patients, as it can help predict potential complications and guide appropriate medical interventions. Family screening and counseling: by identifying the genetic variant in the affected individual, the study demonstrates the utility of genetic testing in identifying asymptomatic family members who may be at risk of developing NF1-related complications. This has implications for genetic counseling and family planning, as it allows for informed decision-making and proactive management of the condition within the family.

## Conclusions

The proband in the current study had NF1 and ACM. This investigation is the first to report ACM in a patient with NF1. Although there is no cure for NF1, genetic tests, such as WES, can identify asymptomatic family members at risk. In addition, cardiac evaluation could assist in helping these patients before cardiac problems develop.

**Accession Number**.

The accession number of the variant in ClinVar is as follows:

NM_001042492.3 (NF1): c.3277G > A (p.Val1093Met): VCV000457638.5.

### Electronic supplementary material

Below is the link to the electronic supplementary material.

Supplementary Material 1

Supplementary Material 2

Supplementary Material 3

Supplementary Material 4

## Data Availability

All data generated or analyzed during this study are included in this published article.
