# Supplementary material for: Whole-exome sequencing revealed a likely pathogenic variant in NF1 causing neurofibromatosis type I and Arrhythmogenic Cardiomyopathy
Source: BMC Cardiovasc Disord. 2024 Apr 23;24:220. doi: 10.1186/s12872-024-03878-z (PMC11036766; doi:10.1186/s12872-024-03878-z)
Supplement: Supplementary file 1 — Supplementary Material 1 [file 12872_2024_3878_MOESM1_ESM.docx]

**Supplements**

**Supplementary video1 and 2:** The four-chamber and mid-level short axis cine sequences showing bi-ventricular dysfunction plus segmental akinesia in the sub-tricuspid region and mid-RV free wall.

**Supplementary video 3:** The right ventricular outflow view cine sequence showing dyskinesia of right ventricular outflow and sub-tricuspid regions.
